# *Echinococcus multilocularis* in foxes and raccoon dogs: an increasing concern for Baltic countries

**DOI:** 10.1186/s13071-016-1891-9

**Published:** 2016-11-29

**Authors:** Guna Bagrade, Gunita Deksne, Zanda Ozoliņa, Samantha Jane Howlett, Maria Interisano, Adriano Casulli, Edoardo Pozio

**Affiliations:** 1 Latvian State Forest Research Institute “Silava”, Rīgas str. 111, LV-2169 Salaspils, Latvia; 2 Institute of Food Safety, Animal Health and Environment “BIOR”, Lejupes str. 3, LV-1076 Riga, Latvia; 3 Istituto Superiore di Sanità, viale Regina Elena 299, 00161 Rome, Italy; 4 WHO Collaborating Centre for the epidemiology, detection and control of cystic and alveolar echinococcosis, ISS, viale Regina Elena299, 00161 Rome, Italy

**Keywords:** *Echinococcus multilocularis*, Red fox, Raccoon dog, Prevalence, Zoonosis, Latvia, Baltic countries

## Abstract

**Background:**

In Europe, the life-cycle of *Echinococcus multilocularis* is predominantly sylvatic, involving red foxes (*Vulpes vulpes*) as the main definitive hosts and rodents such as muskrats and arvicolids as intermediate hosts. The parasite is the etiological agent of human alveolar echinococcosis, a malignant zoonotic disease caused by the accidental ingestion of eggs shed by definitive hosts in their faeces. The aims of this study were to investigate the prevalence of *E. multilocularis* in red foxes and raccoon dogs (*Nyctereutes procyonoides*) and to study the environmental factors favouring the perpetuation of the parasite in Latvia.

**Methods:**

A total of 538 red foxes and 407 raccoon dogs were collected across Latvia from 2010 to 2015. The sedimentation and counting technique was used for collecting *E. multilocularis* adult worms from fox and raccoon dog intestines*.* The morphological identification of the parasite was confirmed by molecular analysis.

**Results:**

The prevalence of *E. multilocularis* was significantly higher in foxes (17.1%; intensity of infection 1–7,050 worms) (*P* < 0.001) than in raccoon dogs (8.1%; intensity of infection 5–815 worms). In foxes, a significant positive correlation (*r*_(10)_ = 0.7952, *P* = 0.001) was found between parasite prevalence and the intensity of infection. A positive relationship (*R*_s_ = 0.900, *n* = 5, *P* = 0.037) between parasite prevalence and precipitation was also observed. In raccoon dogs, a significant negative relationship (*F*_(1,8)_ = 9.412, *P* = 0.015) between animal density and parasite prevalence, and a significant positive relationship (*F*_(1,8)_ = 7.869, *P* = 0.023) between parasite prevalence and agricultural land cover, were detected.

**Conclusions:**

The results of this study confirm the red fox as the most important definitive host of *E. multilocularis* and, consequently, as the main target for control programmes in the Baltic countries. Raccoon dogs seem to play a secondary role in the life-cycle of *E. multilocularis* within the investigated European region.

**Electronic supplementary material:**

The online version of this article (doi:10.1186/s13071-016-1891-9) contains supplementary material, which is available to authorized users.

## Background

Alveolar echinococcosis (AE) is a human disease caused by the metacestode stage of *Echinococcus multilocularis* and is of considerable public health importance in Europe due to the severity of the disease, which leads to death in untreated patients [1, 2]. According to the Centre for Disease Prevention and Control of Latvia (www.spkc.gov.lv), 146 human cases of echinococcosis were reported in Latvia since 2000. From 1999 to 2010, 14 out of the 100 recorded echinococcosis cases were identified as AE, a figure which represents 13% of all the registered echinococcosis infections in Latvia [3, 4].

In Europe, *E. multilocularis* is transmitted predominantly through a wildlife cycle involving primarily the red foxes (*Vulpes vulpes*) as definitive hosts and rodents of the family Arvicolidae as intermediate hosts [5, 6]. Historically, endemic regions for *E. multilocularis* in Europe consisted of a ‘core’ area comprising regions of southern Germany, eastern France, north-central Switzerland, and western Austria [6, 7]. Since the 1990s, broad epidemiological studies showed the endemic area of the parasite to include regions of 17 European countries [7–11] and *E. multilocularis* has been documented in wildlife from Lithuania [12], Estonia [13] and Latvia [14].

The increase in the number of foxes and the colonization of urban and peri-urban areas have been documented for several European countries [15]. Since synanthropic foxes are reservoir hosts of zoonotic pathogens, it is important to understand the factors influencing the transmission of zoonotic parasites including *E. multilocularis* [16]. In recent years, foxes colonized 33 of 47 Estonian towns [17] and *E. multilocularis* was detected in 7.1% of urban foxes [18]. In the city of Riga (capital of Latvia), foxes were mainly recorded in forest parks, meadows, private gardens and on Zakusala island with an increase of their number towards the periphery of the city [19]. Consequently, contamination of the environment with fox faeces could be higher in peri-urban areas and, therefore, these areas hypothetically could be more crucial than rural or highly urbanized areas for parasite transmission [15, 20].

According to the hunting regulations in Latvia, red foxes and raccoon dogs can be hunted all year round with no limitations. The population of raccoon dogs in Latvia is stable, whereas the red fox population has decreased in recent years (State Forest Service, unpublished data).

The aims of the present study were to determine the prevalence of *E. multilocularis* infection in red foxes and raccoon dogs and to investigate environmental factors associated with the occurrence of the parasite in Latvia.

## Methods

### Sample collection and parasitological analysis

Animal carcasses were collected from October to January within the Latvian State programme for the Control and Eradication of Rabies. From 2010 to 2014, animals were collected from the entire Latvian territory; whereas in 2014–2015, animals originated from the eastern part of Latvia bordering the Russian Federation and Belarus [21]. A total of 538 and 407 rabies-free red foxes and raccoon dogs, respectively, were included in this study.

For safety reasons, the whole intestine was frozen at −80 °C for 1 week. Adult worms of *E. multilocularis* were collected by the sedimentation and counting technique using two sieves of 1 mm mesh and 150 μm mesh [22, 23]. *Echinococcus multilocularis* worms were morphologically identified [24] and preserved in 96% ethanol for further analyses.

### Molecular identification

To confirm the morphological identification, 114 worms (82 from red foxes and 32 from raccoon dogs) were tested by molecular analysis according to previously published protocols [25, 26]. Briefly, worms were washed three times in phosphate-buffered saline at pH 7.2 and DNA from individual worms was extracted using the Wizard Magnetic DNA Purification System for Food (Promega, Madison, WI, USA) according to the manufacturer’s instructions. DNA was used as template in a two-step PCR assay. In the first step, DNA from individual worms was amplified using a multiplex-PCR [25] to distinguish *E. multilocularis* from *E. granulosus* and from *Taenia* spp. This multiplex assay was designed to amplify a 395 bp fragment of NADH dehydrogenase subunit 1 (*nad*1) gene of *E. multilocularis* and 117 bp and 267 bp of the small subunit of ribosomal RNA (rrnS) gene of *E. granulosus* and *Taenia* spp., respectively. All samples were tested in 30 μl amplification reaction mixtures containing primers Cest1, Cest2, Cest3, Cest4 and Cest5 as previously described [25]. In the second step, conventional PCR and sequencing targeting mitochondrial cytochrome *c* oxidase subunit 1 (*cox*1) gene were performed on positive amplicons following a previously published protocol [27] with some modifications [26]. This approach was used to confirm species identification. Briefly, the PCR consisted of 38 cycles with a denaturation (30 s at 94 °C), a hybridization (30 s at 55 °C) and an elongation (30 s at 72 °C) step for each cycle. For each PCR reaction, 2 μl of DNA was added to 48 μl of the reaction mixture (GoTaq Master Mix; Promega, Madison, WI, USA), which contained 20 pmol of each primer and 1 unit of Taq polymerase. For the detection of the PCR amplicons, PCR products were separated by capillary electrophoresis (QIAxcel, QIAGEN, Hilden, Germany) using the high resolution kit of the 0 M500 method (QIAGEN, Hilden, Germany). PCR products were purified using a Wizard SV Gel and PCR Clean-Up System (Promega, Madison, WI, USA). Forward and reverse sequencing was carried out on a capillary DNA sequencer ABI PRISM 3100 Genetic Analyzer using the BigDye terminator cycle sequencing kit (Applied Biosystems, Foster City, CA, USA). BLAST algorithm (http://www.ncbi.nlm.nih.gov/BLAST/) was used to compare nucleotide sequences generated in this study with those present on the NCBI database (http://www.ncbi.nlm.nih.gov).

### Data analysis

The age group classified as juvenile or adult by the dental wear was known for 377 foxes (75 juveniles, 302 adults) and 263 raccoon dogs (61 juveniles, 202 adults). Since the gender was only determined for very few animals, it was no longer taken into account for the analysis.

The mean annual temperature and mean annual precipitation data were obtained from the Latvian Environment, Geology and Meteorology Centre (www.meteo.lv, on a request). Information on land cover was acquired from the State Forest Service (SFS) (www.vmd.gov.lv, on a request) and Central Statistical Bureau of Latvia (http://www.csp.gov.lv, on a request). The SFS is a surveillance authority inspecting forest management and hunting. It governs a network of local game administrative units and collects annual hunting records (reference period from 1st April to 31st March) from these game units. Animal density was calculated using data of hunted animals per reference period at the scale of game administrative unit territory. Therefore, data on investigated/ infected animals were presented at the same scale.

The prevalence (with 95% confidence interval) of *E. multilocularis* and the mean intensity of infection were determined. Both Pearson’s and Spearman’s rank correlation tests, Fisher’s exact test, Mann-Whitney *U*-test and linear regression were used for statistical comparison. Statistical analyses were performed using SPSS Statistics Version 20 [28].

## Results

*Echinococcus multilocularis* was significantly more prevalent in red foxes (17.1%; *P* < 0.001) (parasite intensity of 1–7,050 worms) than in raccoon dogs (8.1%) (parasite intensity of 1–815 worms) (Table 1). There was a significant positive correlation (*r*_(10)_ = 0.7952, *P* = 0.001) between parasite prevalence and intensity of infection for foxes; however, no such correlation was detected for raccoon dogs. The proportion between investigated and hunted animals in the whole territory was about 1% (Additional file 1: Table S1).

**Table 1:** Prevalence and intensity of *Echinococcus multilocularis* worms in red foxes and raccoon dogs in Latvia from 2010 to 2015

Year	Red foxes				Raccoon dogs			
	No. of infected/tested animals	Prevalence (%) (95% CI)	Mean intensity (95% CI)	Intensity range	No. of infected/tested animals	Prevalence (%) (95% CI)	Mean intensity (95% CI)	Intensity range
2010/2011	42/177	23.7 (17.5–30.0)	307.8 (0–636.8)	1–7,050	5/98	5.1 (0.7–9.5)	184.2 (0–493.3)	19–815
2011/2012	18/113	15.9 (9.2–22.7)	172.8 (45.1–300.5)	1–902	8/96	8.3 (2.8–13.7)	34.8 (8–61.5)	6–123
2012/2013	12/58	20.7 (10.3–31.1)	54.7 (0–157.5)	5–317	3/56	5.4 (0–11.3)	28.7 (5.5–51.9)	8–49
2013/2014	5/76	6.6 (1.0–12.2)	117.8 (0–250.8)	2–371	1/56	1.8 (0–5.3)	72	72
2014/2015^a^	15/114	13.1 (6.9–19.4)	1,020.2 (0–2132.8)	1–6,748	16/101	15.8 (8.7–23.0)	152.6 (5.5–51.9)	1–810
Total	92/538	17.1 (13.9–20.3)	375.1 118–632.2	1–7,050	33/407	8.1 (5.5–10.8)	115.1 (44.8–185.4)	1–815

^a^In 2014/2015 material was collected only in eastern Latvia (border territory with Russian Federation and Belarus)

**Fig. 1:**
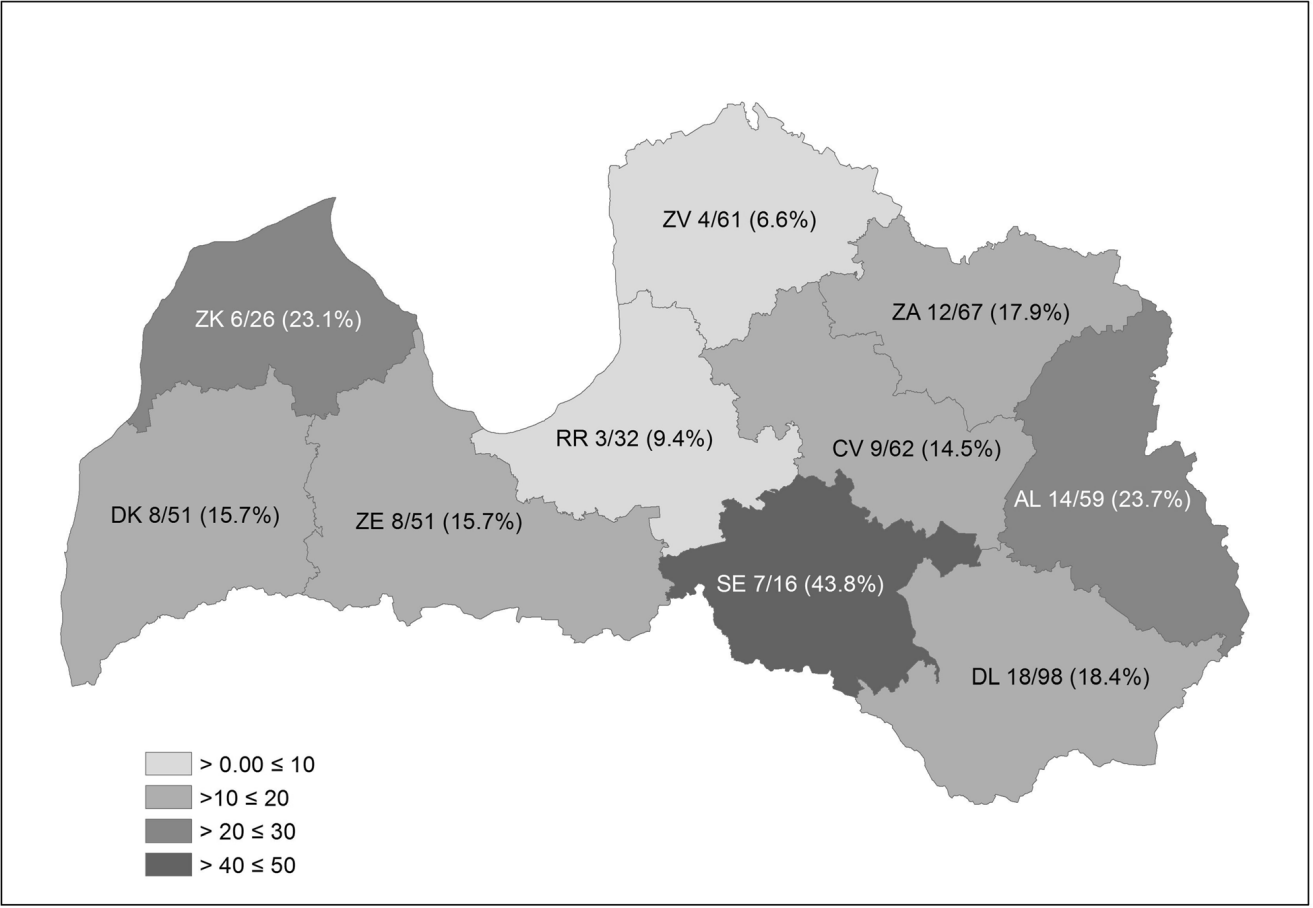
*Echinococcus multilocularis* prevalence in foxes of Latvia from 2010 to 2015. Game administrative units: DK, Dienvidkurzeme; ZK, Ziemeļkurzeme; ZE, Zemgale; RR, Rīgas reģionālā; SE, Sēlija; DL, Dienvidlatgale; AL, Austrumlatgale; ZA, Ziemeļaustrumu; CV, Centrālvidzeme; ZV, Ziemeļvidzeme. Numbers indicate positive/tested (%) animals per game administrative unit

**Fig. 2:**
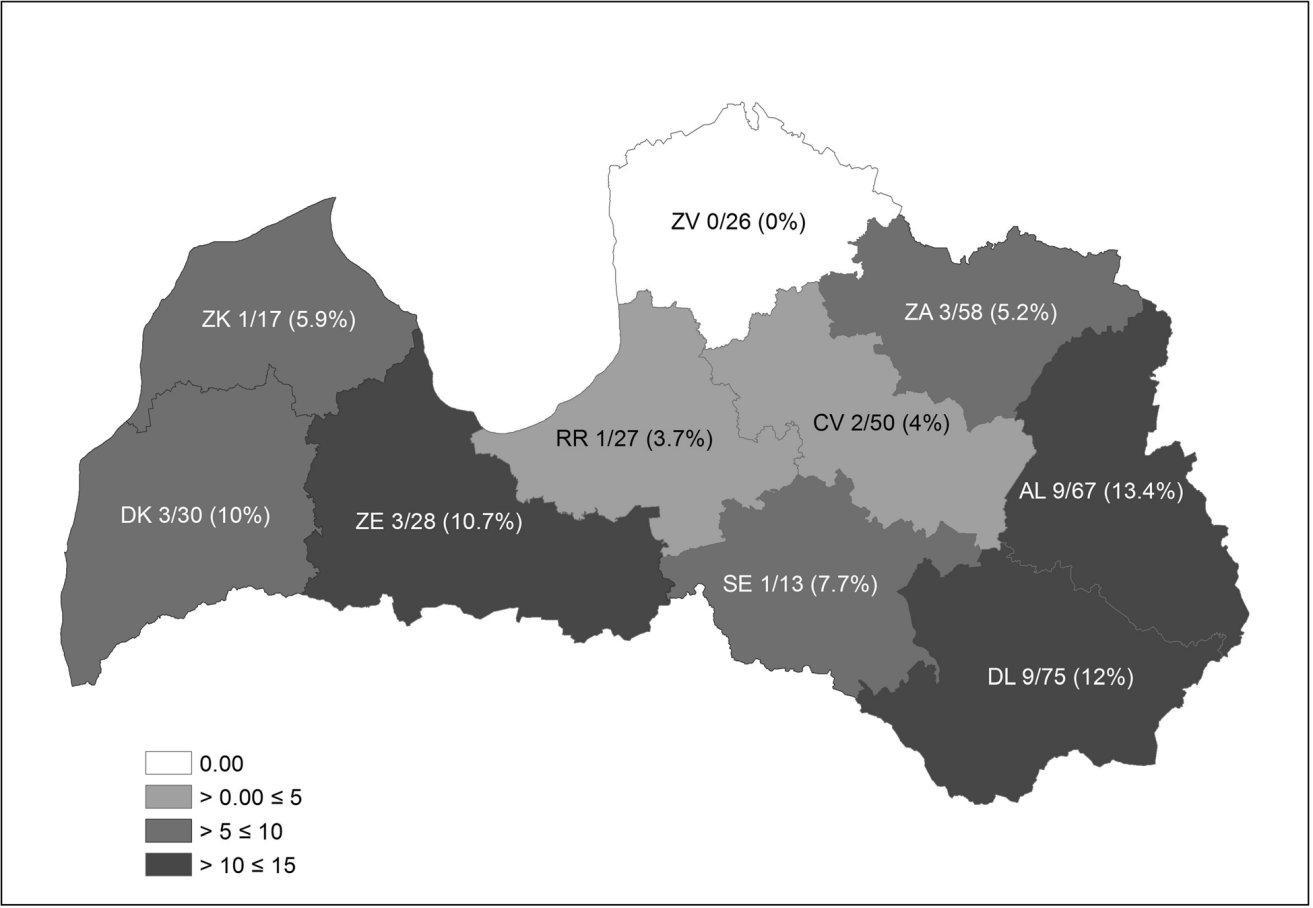
*Echinococcus multilocularis* prevalence in raccoon dogs of Latvia from 2010 to 2015. Game administrative units: DK, Dienvidkurzeme; ZK, Ziemeļkurzeme; ZE, Zemgale; RR, Rīgas reģionālā; SE, Sēlija; DL, Dienvidlatgale; AL, Austrumlatgale; ZA, Ziemeļaustrumu; CV, Centrālvidzeme; ZV, Ziemeļvidzeme. Numbers indicate positive/tested (%) animals per game administrative unit

**Fig. 3:**
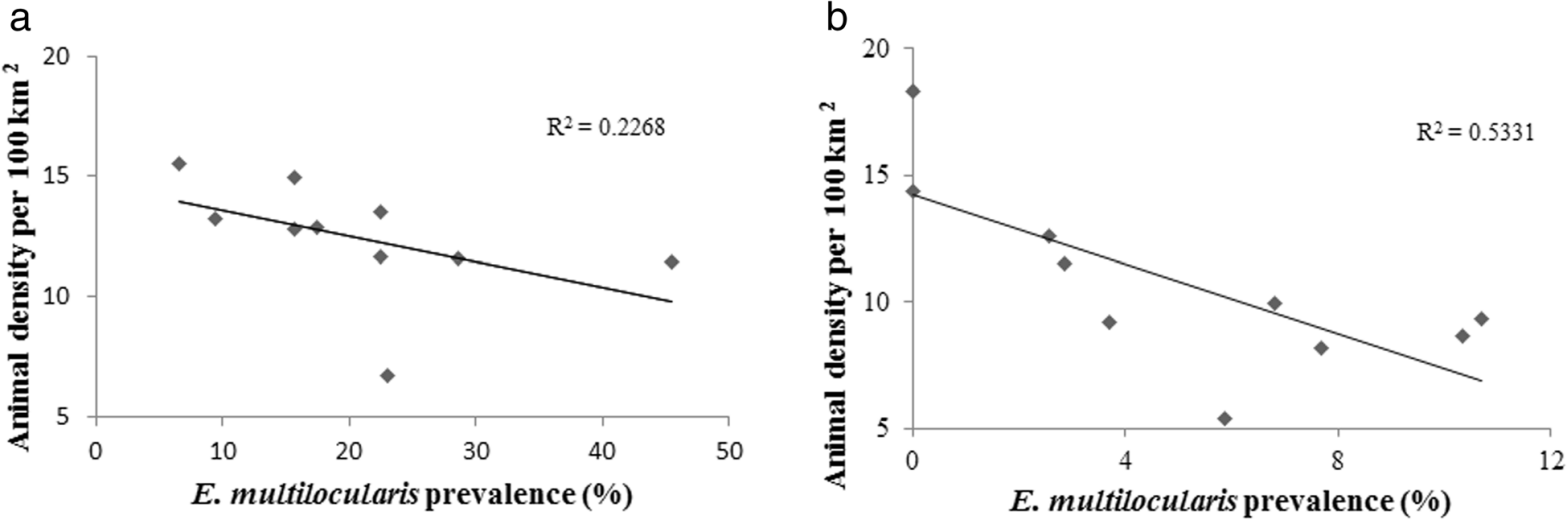
Correlation between the *Echinococcus multilocularis* prevalence and the animal population density for foxes (**a**) and raccoon dogs (**b**)

Infected foxes were found to occur across the whole territory of Latvia (Fig. 1, Additional file 1: Table S1). Prevalence did not change significantly between the eastern and western part of Latvia. Infected raccoon dogs were found in all game administrative units except one (Fig. 2, Additional file 1: Table S1). There was no significant correlation between the total number of harvested animals and the infection rate for both species by game administrative units. The linear regression between raccoon dog density and *E. multilocularis* prevalence showed a significant negative relationship (*F*_(1,8)_ = 9.412, *P* = 0.015) (Fig. 3). The same tendency was observed for foxes although it was not statistically significant (*F*_(1,8)_ = 2.115, *P* = 0.133) (Fig. 3).

There was no statistically significant difference between the age of host species and parasite prevalence (Fisher’s exact test), or between the age and parasite intensity (Mann-Whitney *U*-test).

**Fig. 4:**
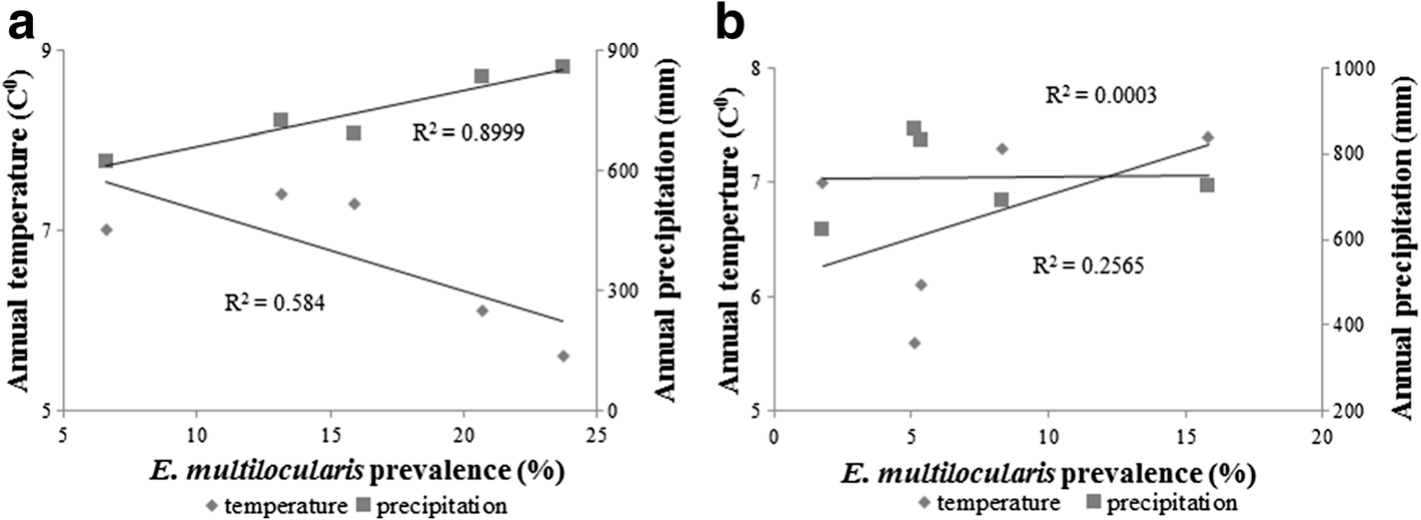
Relationship between climate factors and *Echinococcus multilocularis* prevalence in foxes (**a**) and raccoon dogs (**b**)

A temporal fluctuation of *E. multilocularis* prevalence was observed for both species in the study period (Table 1). In foxes, there was a positive relationship (*R*_s_ = 0.900, *n* = 5, *P* = 0.037) between *E. multilocularis* prevalence and precipitation and a negative but non-significant relationship (*R*_s_ = -8.66, *n* = 5, *P* = 0.058) between the prevalence and the environmental temperature (Fig. 4). No apparent relationship was observed between prevalence and environmental temperature (*R*_s_ = 0.296, *n* = 5, *P* = 0.628) or prevalence and precipitation (*R*_s_ = 0.154, *n* = 5, *P* = 0.805) for raccoon dogs (Fig. 4).

**Fig. 5:**
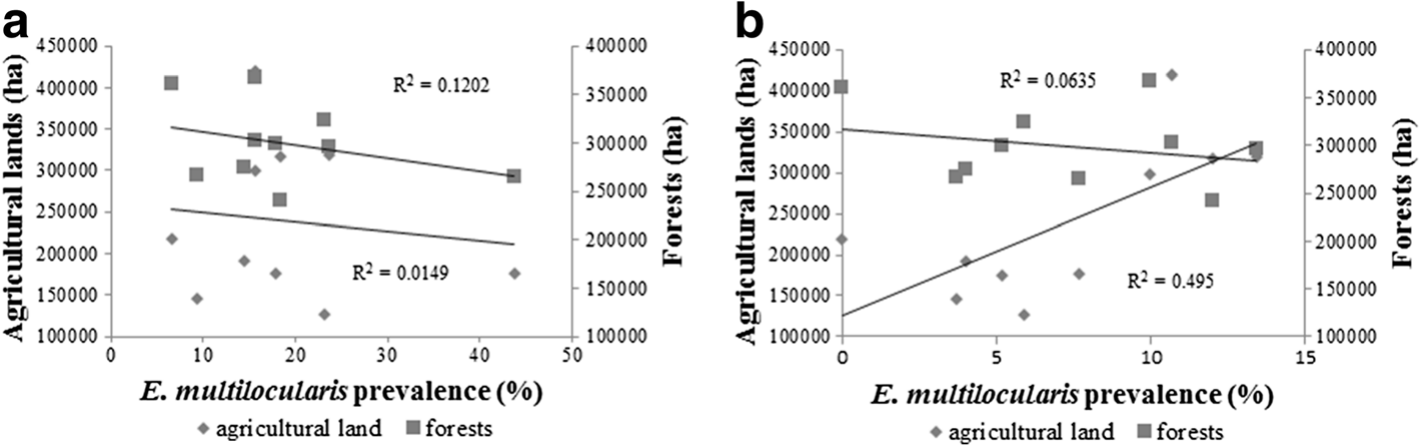
Relationship between land cover and *Echinococcus multilocularis* prevalence in foxes (**a**) and raccoon dogs (**b**) within each game administrative unit

A negative but non-significant relationship was found between *E. multilocularis* prevalence in foxes and the amount of forest or agricultural land within each game administrative unit (*R* ^2^ = 0.120, *F*_(1,8)_ = 0.100, *P* = 0.759 and *R* ^2^ = 0.015, *F*_(1,8)_ = 0.898, *P* = 0.371, respectively). In raccoon dogs, there was no significant relationship between prevalence and forest cover (*R* ^2^ = 0.064, *F*_(1,8)_ = 0.605, *P* = 0.459), but a statistically significant positive relationship (*R* ^2^ = 0.495, *F*_(1,8)_ = 7.869, *P* = 0.023) was detected between prevalence and agricultural land (Fig. 5).

*Echinococcus multilocularis* worms were successfully identified in 69 isolates (82.1%). Out of the 15 (17.9%) isolates, which did not show any molecular amplification for *E. multilocularis*, 10 showed a positive reaction for Taeniidae. In three of these animals, *E. multilocularis* and *Taenia* sp. worms were morphologically identified.

## Discussion

The prevalence and distribution of *E. multilocularis* in foxes in Latvia has been investigated since 2003. In the study of Bagrade et al. [14], 16 out of 45 tested animals (35.6%) were infected, and most of the positive foxes originated from western Latvia. Poļakova [29] detected *E. multilocularis* in 19.1% (*n* = 42) of foxes collected from only three game administrative units of Latvia. The results of the present study show no significant difference of *E. multilocularis* prevalence between the eastern and western part of the country. The highest prevalence of *E. multilocularis* in foxes was detected in three game administrative units, two of which were in the eastern part (DL and AL, Fig. 1) and one in the western part (SE) of Latvia. However, the high prevalence in SE could have been influenced by the small sample size. The 17.1% prevalence of *E. multilocularis* found in foxes in the present study is lower than in Estonia (29.4%) and significantly lower than in Lithuania (58.7%; *P* < 0.001). However, the sample size in the studies from Estonia and Lithuania was lower than in the present study, 17 and 269, respectively [13, 30, 31].

Previous studies on *E. multilocularis* in the raccoon dog population of Latvia showed a prevalence ranging from 5.3% (*n* = 19, from different regions of the country; [32]) to 14.3% (*n* = 42, from three game administrative units; [29]). During the current study, *E. multilocularis* was not detected in raccoon dogs hunted in one game administrative unit (ZV, Fig. 2, Additional file 1: Table S1), although this parasite had been previously reported in this territory [29].

In other European countries (Estonia, Lithuania, Poland and western part of Germany), *E. multilocularis* prevalence in raccoon dogs ranged from 1.6 to 8.2% [31, 33–35]. The present data are in agreement with previous studies and confirm the susceptibility of the raccoon dog to *E. multilocularis* infection, although its role as a final host of the life-cycle may be of less importance than that of the red fox. The susceptibility of the raccoon dog to *E. multilocularis* infection has been also confirmed by experimental infections [36]. The feeding behaviour of the raccoon dog may partially explain the lower prevalence and worm intensity detected in this host species. In Denmark, where *E. multilocularis* was detected in foxes, none of the raccoon dogs examined (*n* = 99) were infected with *E. multilocularis*. These data highlight the importance of the feeding behaviour; in fact, amphibian-transmitted parasite species were more prevalent in raccoon dogs than in foxes, in which rodent-transmitted parasites prevailed [37]. In Estonia, a difference between the raccoon dog diet and the fox diet was observed in the cold period of the year, revealing anthropogenic plants and carrions as the most important food sources for raccoon dogs, while foxes consumed significantly more arvicolid rodents [38].

According to Hegglin et al. [39], some raccoon dog behavioural traits could affect the parasite cycle. The use of latrines renders the raccoon dog epidemiologically less important compared to red foxes for transmission and perpetuation of *E. multilocularis* life-cycle. Moreover, the raccoon dog, as well as other wild canids such as the golden jackal (*Canis aureus*) and the wolf (*Canis lupus*), can act as definitive hosts, but there is no evidence that they can maintain the life-cycle of the parasite in the absence of the red fox [40]. Meanwhile, studies from western Germany [34] and Estonia [35] indicate an increasing importance of the raccoon dog as a definitive host, particularly because they are becoming widespread and have well-established populations, and share the same areas with foxes.

In recent years, several biotic and abiotic factors (e.g., landscape change composition and use, fox urbanization, wildlife introduction, changing human behavioural attitudes toward foxes, climate change, and host-parasite population dynamics) were suggested as explanations behind the increasing risk of *E. multilocularis* transmission [41]. These factors and their interactions change across regions and even between different ecosystems of the same area [16, 42].

The results of this study show a statistically significant negative relationship between raccoon dog density and *E. multilocularis* prevalence (Fig. 3). This finding may be influenced by the use of latrines, which increase the probability that rodents acquire the infection only in restricted areas, irrespective of the raccoon dog density. In contrast, in eastern France, a positive correlation was found between fox density and *E. multilocularis* prevalence; indeed, a decreased prevalence was associated with a reduction in fox numbers [43]. According to Karamon et al. [44], an increasing animal population is not always an absolute factor determining a higher prevalence of the parasite.

This study demonstrated a correlation between *E. multilocularis* prevalence and worm intensity in foxes, i.e., the increased prevalence was associated with an increased worm burden. Several studies revealed that the distribution of *E. multilocularis* biomass was highly aggregated; therefore, a few highly infected foxes are responsible for most of the environmental egg contamination [15, 45, 46].

No statistically significant age-dependent difference between *E. multilocularis* prevalence or intensity of infection in either foxes or raccoon dogs was detected. However, the age was known only for a small number of animals in our study.

In Latvia, a strong west-east gradient characterizes both the continental climate and the distribution of precipitation, and a south-north configuration of uplands and lowlands plays an important role in climate differentiation when moving away from the sea to the inland [47]. Comparing the fluctuation of *E. multilocularis* prevalence with climate factors in Latvia, results showed a slight tendency towards an increase in *E. multilocularis* prevalence in the fox population in the years characterised by lower temperatures and higher precipitation (Fig. 4). Interestingly, when the parasite prevalence in the fox population was increasing, the opposite tendency was observed in the raccoon dog population and vice versa. The only exception was during 2013/2014 period, when a dramatic decrease in *E. multilocularis* prevalence was observed in both host species. The temporal fluctuation of *E. multilocularis* prevalence in raccoon dogs could be related to sampling bias (more animals sampled from a single clan) influenced by animal behavioural traits (use of latrines). The effect of climate on the prevalence and burden of *E. multilocularis* was shown in a long-term study in Slovakia, where the highest parasite prevalence in foxes was detected in the years with a lower mean annual air temperature and a higher mean annual rainfall [48–50]. Mean annual temperature and annual precipitations were also noted as the major determinants of the spatial distribution of *E. multilocularis* in Hungary [51]. However, studies in Poland did not show a strict correlation between the annual rainfall and the parasite prevalence [44].

In the present study, the comparison between parasite prevalence in foxes and raccoon dogs with land cover (forest and agricultural land) showed a statistically significant difference only for the raccoon dog and agricultural land (Fig. 5). In Poland, a weak negative correlation was detected between *E. multilocularis* prevalence in foxes and agricultural land, and a positive correlation between *E. multilocularis* prevalence in foxes and forest cover [44].

*Echinococcus multilocularis* has been recorded in wolves from Latvia in 2003 [52]. In the Ziemeļkurzeme game administrative unit, one of the areas with the highest parasite prevalence in the fox population, *E. multilocularis* was detected in all three canid species (fox, raccoon dog and wolf). This might suggest that environmental conditions within this area favour the *E. multilocularis* life-cycle.

## Conclusions

The significant difference in the prevalence of *E. multilocularis* between foxes and raccoon dogs, found in this study, is in agreement with other studies carried out within the Baltic region [31]. The significant correlation between parasite prevalence and the intensity of infection in foxes recorded here provides strong evidence to the role of the red fox in the contamination of the environment with *E. multilocularis* eggs. However, the importance of the raccoon dog as a definitive host, as suggested by other studies [34, 35], should not be underestimated in the presence of a stable fox population and a high *E. multilocularis* prevalence.

## Additional file

Additional file 1: Table S1. Prevalence of *Echinococcus multilocularis* in foxes and raccoon dogs, mean density of animals, hunting bags and ratio of sampled animals out of hunted during 2010–2014 on a scale of game administrative units in Latvia. (DOCX 24 kb)

## Abbreviations

AE: Alveolar echinococcosis
*cox*1: Cytochrome *c* oxidase subunit 1
*nad*1: NADH dehydrogenase subunit 1
rrnS: Small subunit of ribosomal RNA
SFS: State Forest Service
