# Supplementary material for: Echinococcus multilocularis in foxes and raccoon dogs: an increasing concern for Baltic countries
Source: Parasit Vectors. 2016 Nov 29;9:615. doi: 10.1186/s13071-016-1891-9 (PMC5129665; doi:10.1186/s13071-016-1891-9)
Supplement: Additional file 1: Table S1. — Prevalence of Echinococcus multilocularis in foxes and raccoon dogs, mean density of animals, hunting bags and ratio of sampled animals out of hunted during 2010–2014 on a scale of game administrative units in Latvia. (DOCX 24 kb) [file 13071_2016_1891_MOESM1_ESM.docx]

**Additional file 1: Table S1.** Prevalence of *Echinococcus* *multilocularis* in foxes and raccoon dogs, mean density of animals, hunting bags and ratio of sampled animals out of hunted during 2010–2014^a^ on a scale of game administrative units in Latvia

| Game administrative unit |  |  | **Foxes** | | | | | **Raccoon dogs** | | | |
| --- | --- | --- | --- | --- | --- | --- | --- | --- | --- | --- | --- |
|  | No. of hunted animals | Mean density of animals per 100 km^2^ | No. of tested/ infected animals | Ratio of sampled animals out of hunted, (%) | Prevalence (%) | No. of hunted animals | Mean density of animals per 100 km^2^ | | No. of tested/ infected animals | Ratio of sampled animals out of hunted, (%) | Prevalence (%) |
| AL | 3192 | 11.6 | 42/12 | 1.3 | **28.6** | 2257 | 8.2 | | 39/3 | 1.7 | **7.7** |
| ZE | 5134 | 15.0 | 51/8 | 1.0 | **15.7** | 3193 | 9.3 | | 28/3 | 0.9 | **10.7** |
| DK | 3785 | 12.8 | 51/8 | 1.4 | **15.7** | 2570 | 8.7 | | 30/3 | 1.2 | **10.0** |
| DL | 4137 | 13.5 | 49/11 | 1.2 | **22.5** | 3050 | 10.0 | | 44/3 | 1.5 | **6.8** |
| CV | 3003 | 12.9 | 46/8 | 1.5 | **17.4** | 2940 | 12.6 | | 39/1 | 1.3 | **2. 6** |
| RR | 3071 | 13.2 | 32/3 | 1.0 | **9.4** | 2138 | 9.2 | | 27/1 | 1.3 | **3.7** |
| SE | 2536 | 11.4 | 11/5 | 0.4 | **45.5** | 3201 | 14.4 | | 8/0 | 0.3 | **0** |
| ZA | 2499 | 11.6 | 40/9 | 1.6 | **22.5** | 2473 | 11.5 | | 35/1 | 1.4 | **2.9** |
| ZK | 1412 | 6.7 | 26/6 | 1.8 | **23.1** | 1137 | 5.4 | | 17/1 | 1.5 | **5.9** |
| ZV | 3860 | 15.5 | 61/4 | 1.6 | **6.6** | 4562 | 18.3 | | 24/0 | 0.5 | **0** |

^a^Excluded last season (2014/2015) due to lack of hunting data of this period and material was collected only in eastern part of Latvia (border territory with Russian Federation and Belarus). Game administrative unit *abbreviations*: DK, Dienvidkurzeme; ZK, Ziemeļkurzeme; ZE, Zemgale; RR, Rīgas reģionālā; SE, Sēlija; DL, Dienvidlatgale; AL, Austrumlatgale; ZA, Ziemeļaustrumu; CV, Centrālvidzeme; ZV, Ziemeļvidzeme
